# Histone Deacetylases Exert Class-Specific Roles in Conditioning the Brain and Heart Against Acute Ischemic Injury

**DOI:** 10.3389/fneur.2015.00145

**Published:** 2015-06-30

**Authors:** Sverre E. Aune, Daniel J. Herr, Craig J. Kutz, Donald R. Menick

**Affiliations:** ^1^Gazes Cardiac Research Institute, Medical University of South Carolina, Charleston, SC, USA

**Keywords:** ischemia-reperfusion injury, histone deacetylase inhibitors, stroke, posttranslational modification, enzymatic crosstalk, preconditioning, postconditioning, reperfusion injury salvage kinase

## Abstract

Ischemia-reperfusion (IR) injury comprises a significant portion of morbidity and mortality from heart and brain diseases worldwide. This enduring clinical problem has inspired myriad reports in the scientific literature of experimental interventions seeking to elucidate the pathology of IR injury. Elective cardiac surgery presents perhaps the most viable scenario for protecting the heart and brain from IR injury due to the opportunity to condition the organs prior to insult. The physiological parameters for the preconditioning of vital organs prior to insult through mechanical and pharmacological maneuvers have been heavily examined. These investigations have revealed new insights into how preconditioning alters cellular responses to IR injury. However, the promise of preconditioning remains unfulfilled at the clinical level, and research seeking to implicate cell signals essential to this protection continues. Recent discoveries in molecular biology have revealed that gene expression can be controlled through posttranslational modifications, without altering the chemical structure of the genetic code. In this scenario, gene expression is repressed by enzymes that cause chromatin compaction through catalytic removal of acetyl moieties from lysine residues on histones. These enzymes, called histone deacetylases (HDACs), can be inhibited pharmacologically, leading to the de-repression of protective genes. The discovery that HDACs can also alter the function of non-histone proteins through posttranslational deacetylation has expanded the potential impact of HDAC inhibitors for the treatment of human disease. HDAC inhibitors have been applied in a very small number of experimental models of IR. However, the scientific literature contains an increasing number of reports demonstrating that HDACs converge on preconditioning signals in the cell. This review will describe the influence of HDACs on major preconditioning signaling pathways in the heart and brain.

## Introduction

Worldwide, 33 million people suffer a stroke each year (1). Ischemia, which can occur in all tissues, is defined as the stress that a tissue experiences when both oxygen and substrate are reduced (2). Stroke, defined as insufficient blood flow to the brain, is one major type of cerebral ischemia and may cause transient to permanent loss of brain function or death (3). In a subset of patients (<15%), stroke occurs as a result of cardiac arrest and cardiac surgery; secondary stroke is, by definition, the result of embolic events originating in the heart (4). In spite of intense research effort directed toward reducing the impact of these conditions, world rates of morbidity and mortality as a result of brain and heart disease continue to rise, most strikingly in developing countries (5). The lack of broadly effective treatment continues to fuel the search for new molecular targets in ischemia-reperfusion injury (IRI). Intriguingly, studies of molecular pathology in the brain and heart are often informed by cancer research. Recent clinical advances have revealed the efficacy of using small molecule inhibitors of histone deacetylases (HDACs) to target malignancy (6). HDACs are enzymes which control signal transduction and gene expression in all cell types (7). Here we review the experimental evidence in support of applying HDAC inhibitors in settings of cerebral and cardiac ischemia, with emphasis on the roles that HDACs play in signaling events that occur as a result of IRI. Importantly, this nascent experimental work indicates that HDAC inhibitors show great promise for treating patients at high risk for stroke or cardiac arrest and for patients electing to receive brain and heart surgeries.

Endogenous tolerance to ischemia can be evoked in the heart and brain (4, 8). Classical ischemic conditioning in a tissue requires the mechanical application of several momentary, sub-lethal reductions in oxygen and substrate delivery, which reduce the injury caused by a more severe ischemic insult (9). Organs subjected to classical ischemic conditioning experience only transient stimulation of endogenous protective mechanisms, which limits application of the conditioning stimulus to shortly before prolonged insult, as in preconditioning a patient of elective surgery, or to shortly afterward, as in postconditioning a patient of out-of-hospital stroke or cardiac arrest. The idea that similar magnitudes of endogenous protection can be evoked pharmacologically is a considerable advancement over the barrier of time-dependence instituted by mechanical ischemic conditioning (10). It follows that drug-evoked conditioning is a promising treatment strategy for patients undergoing elective brain or heart surgery, or those at high risk of stroke or cardiac arrest (11).

Prevention of cell death is the primary effect of ischemic conditioning (12, 13). Many forms of ischemic conditioning (including classical, pharmacological, and remote conditioning) converge on signaling pathways that either inhibit cell death or activate endogenous cell survival maintenance programs. Once active, cell survival programs improve endoplasmic reticulum stabilization (14), increase expression of antioxidant enzymes (SOD2; catalase) (15), inhibit endogenous cell death programs (Bax/Bad/Bcl2; c-jun) (16), activate transcription of genes for repair enzymes (Hsp70; HIF-1-alpha) (17, 18), and stimulate autophagic flux (HDAC6) (19).

Acute adaptation to ischemia through conditioning stimuli is mediated by protein posttranslational modifications (PTMs) (20, 21). Historically, protein phosphorylation has been the most rigorously characterized of the PTMs in settings of experimental ischemic conditioning. These include the reperfusion injury salvage kinases (RISK) pathways of Raf/MEK/ERK1/2 and PI3K/Akt/eNOS, the JAK/STAT transcriptional pathways and the calcium-responsive PKC pathways (22–25). However, accumulating evidence shows that protein acetylation also plays major roles in regulating cell survival through ischemic conditioning (7).

## Histone Deacetylases Regulate Cell Fate in Cerebral Ischemia

Histone deacetylases are a class of epigenetic enzymes that have come under recent intense scrutiny as pharmacological targets for patients suffering stroke. HDACs remove acetyl moieties from ε-amino groups of lysine residues on histones and non-histone proteins (25). Deacetylation of histones enhances chromatin compaction, which renders DNA less available for binding by regulatory factors leading to repression of gene expression (26, 27). In this function, HDACs exert classical transcriptional control. This process is reversible via enzymatic histone acetyltransferase (HAT) activity. The zinc-dependent HDACs have been divided into classes based on homology to yeast transcriptional repressors. Class I comprises HDACs 1, 2, 3, and 8; class IIa comprises 4, 5, 7, and 9; class IIb comprises 6 and 10; and class IV comprises HDAC11 (7). Class III, called sirtiuns, are NAD^+^-dependent and will not be discussed in this review, but have been expertly reviewed elsewhere (28). HDAC enzymes are widely expressed in rodent brains, and are localized to specific cellular compartments in isoform-specific patterns (29, 30). Class I HDACs are generally restricted to the nucleus where they impose transcriptional control, whereas class IIa HDACs transit the nuclear membranes and enter the cytoplasm in a process mediated by phosphorylation (31). Furthermore, HDAC6 is primarily, though not constitutively, cytoplasmic (19).

As mentioned above, HDACs deacetylate both histone and non-histone proteins. When HDACs deacetylate histones and repress gene transcription, cell survival is impacted after several hours and days on time scales necessary for protein expression (7). Non-histone deacetylation is another element of this complex code of enzymatic crosstalk, which is distinct from direct inhibition of gene expression by histone deacetylation. Importantly, the acetylation state of a given metabolic signaling factor may mediate its phosphorylation, methylation, and ubiquitination state, thereby determining its subcellular location, activation, or degradation, with immediate implications for cell survival in the seconds to minutes following the insult (32). The acetylation state of transcription factors, co-activators, and co-repressors can regulate their activity (33). Decoding the complex patterns of HDAC enzymatic crosstalk will enhance our understanding of histone and non-histone protein lysine deacetylation and its impact on the survival of cells under ischemic stress (34).

Importantly, experimental cerebral ischemia causes upregulation of class I/IIb HDAC expression, which possibly implicates them in ischemic pathology (29). For example, HDAC1 must exist in complex with HDAC3 to promote apoptosis in cerebellar granule neurons; the toxic effects of HDAC1:HDAC3 association were mitigated by activation of PI3K/Akt signaling (35). Cortical neurons transfected with HDAC3 or HDAC6 shRNA each exhibited decreased apoptosis when exposed to prolonged oxygen–glucose deprivation (30). Furthermore, HDAC2 mutant mice exhibited reduced retinal degeneration following ischemia (36). In response to these findings and many others, a variety of pharmacological HDAC inhibitors have been developed and applied in animal models of cerebral ischemia. Results from these studies have revealed that HDAC inhibitors can pharmacologically condition the neuron against ischemic injury through de-repression of transcription (37, 38). While these studies suggest that pharmacological inhibition of class I/IIb HDAC isoforms (so-called “pan-inhibitors”) promote survival of stressed neurons, the evidence reviewed below indicates that the catalytically inactive IIa isoforms may be beneficial to cell survival from IRI.

## Small Molecule HDAC Inhibitors Condition the Brain Against Ischemic Injury

Reduction in infarct volume and prevention of apoptosis are the most obvious physiological effects of class I/IIb HDAC inhibitors in experimental cerebral ischemia (see Table 1). The molecular mediators are numerous but consensus is forming around certain cellular processes: modulation of apoptotic intermediates (caspases, Bcl-2), stabilization of the cellular stress response (Hsp-70, EIF-2α, CHOP), transcription of oxygen-responsive enzymes (HIF-1α, Nrf2), regulation of calcium handling (BDNF, CREB), and activation of survival kinase cascades (Akt, ERK, AMPK, p21).

**Table 1:** **Physiological effects of HDAC inhibitors in experimental models of stroke**.

Reference	Stroke model	Treatment	Treatment time	Molecular target	Acetylated protein	Physiological effect
(36)	Mouse retinal I/R	HDAC2^+/−^			Ac-histone H3	↓Apoptosis
(39)	Rat pup RCAo + 1 h hypoxia	VPA 200 or 400 mg/kg/day	Post for 5 days			↓Neuronal apoptosis
(40)	Rat MCAo (1 h) with reperfusion	VPA 200 mg/kg/day	Post for 14 days	↑HIF-1α, VEGF, MMP-2/9	Ac-histone H3; Ac-histone H4	↓Brain infarction
(41)	Rat pMCAo	VPA 100 mg/kg/day	Post for 7 days	↑GLT-1	Ac-histone H4	↓Brain infarction; ↓neuronal apoptosis
(43)	Rat pMCAo	VPA 300 mg/kg/12 h × 2	Post for 1 or 2 days	↑HSP-70, p53; ↓iNOS	Ac-histone H3	↓Brain infarction
(43)	Rat pMCAo	SB 300 mg/kg/12 h × 2	Post for 1 or 2 days	↑HSP-70, COX-2, p-Akt	Ac-histone H3	↓Brain infarction
(43)	Rat pMCAo	TSA 0.5 mg/kg/12 h × 2	Post for 1 or 2 days	↑HSP-70, Bcl-2, p-Akt	Ac-histone H3	↓Brain infarction
(44)	Rat MCAo (1 h) with reperfusion	VPA 300 mg/kg/12 h × 2	Post for 1 or 2 days	↑HSP-70; ↓active caspase-3	Ac-histone H3	↓Brain infarction
(45)	Rat (optical nerve crush)	VPA 300 mg/kg/12 h × 2	Post for 5 or 8 days	↑CREB DNA binding, p-ERK; ↓active caspase-3		↓Retinal ganglion cell death; ↑axonal regeneration
(46)	Rat retinal I/R	VPA 300 mg/kg/day	Pre for 1 day and post for 7 days	↑GRP78; ↓active caspase-12, CHOP	Ac-histone H3	↓Retinal ganglion cell death; ↓ER stress-mediated apoptosis
(47)	Heat shock (42°C) 1 h in cultured rat cortical neurons	VPA 0.25–1.0 mM	Post for 1 day	↑HSP-70; ↓active caspase-3	Ac-histone H3K9/14; Ac-Sp1	
(53)	Rat pMCAo	SB 1200 mg/kg	Pre for 1 day and post for 30 min	↑p21	Ac-histone H4	↓Brain infarction
(42)	Rat pMCAo	SB 300 mg/kg day	Post for 14 days	↑BDNF, p-CREB, GFAP		↑Cell proliferation, migration, differentiation
(56)	Mouse MCAo + hypoxia	4-PBA 40 or 120 mg/kg/day	Pre for 3 days or post for 3 days	↓Active caspase-12, p-EIF-2α, CHOP		↓Brain infarction; ↓neuronal apoptosis; ↓ER stress-mediated apoptosis
(58)	Mouse pMCAo	Vorinostat 50 mg/kg x 2	Post at 0 h and 6 h	↑HSP-70, Bcl-2, p-Akt	Ac-histone H3 K18	↓Brain infarction
(59)	OGD (3 h) with reperfusion (2 h) in mouse cultured cortical neurons	Entinostat 0.1, 0.5, or 1 μM	Post for 2 h	↑p-AMPK, Bcl-xL promoter Ac; ↓Bim promoter Ac	Ac-NF-kB p50 K310; Ac-histone H3 K9/18	↓Neuronal apoptosis
(59)	Mouse MCAo (1 h) with reperfusion	Entinostat 20 or 200 μg/kg	Post at 1, 3, 5, or 7 h	↑Bcl-xL promoter Ac; ↓Bim promoter Ac	Ac-histone H3 K9/18	↓Brain infarction
(49)	Mouse MCAo (1 h) with reperfusion	TSA 1 or 5 mg/kg/day	Post for 14 days	Gelsolin	Ac-histone H4	↓Brain infarction
(49)	OGD for 90 or 150 min in mouse cultured cortical neurons	TSA 300 nM	Pre for 12 h			↓[Ca2+]^i^, ↑ΔΨ
(50)	Mouse pMCAo	TSA 1 or 5 mg/kg/day	Post at 0 and 6 h			↓Brain infarction
(50)	OGD for 150 min in mouse cultured cortical neurons	TSA 3, 10, or 30 ng/mL	Pre for 1 h	↑Nrf2:ARE binding, NQO1, HO1		↓Neuronal apoptosis
(51, 52)	Rat retinal I/R	TSA 2.5 mg/kg/12 h	Post for 3 days	↑TNF-α	Ac-histone H3	↓Apoptosis

*RCAo, right carotid artery occlusion; pMCAo, permanent carotid artery occlusion; ONC, optical nerve crush; Ac, acetylated; OGD, oxygen–glucose deprivation*.

Administration of the mood stabilizer and weak HDAC inhibitor valproic acid (VPA) after permanent right carotid artery occlusion prevented neuronal apoptosis in a dose-dependent manner in neonatal rats (39). Rats treated with VPA during middle cerebral artery occlusion (MCAo) exhibited reduced infarct volume, enhanced angiogenesis (40), neurogenesis (41, 42), and reduction of monocyte infiltration (43). These effects were correlated with increased transcription of Hsp70 (43, 44), HIF1-alpha and MMP-2/9 (40), or GLT-1, a transporter protein, which accelerates clearance of glutamate in damaged gray and white matter (41). VPA protected retinal ganglion cells from ischemia-reperfusion (IR) through reduction of mitochondria-mediated apoptosis (45) and endoplasmic reticulum stress-induced apoptosis (46), and enhanced Hsp70 promoter acetylation in cortical neurons through inhibition of a class I HDAC (47). The potent hydroxamate trichostatin A (TSA) decreased infarct volume in rodents given MCAo, which depended on induction of Hsp70, Bcl-2, and Akt phosphorylation, or on gelsolin, an essential regulator of actin homeostasis (43, 48, 49). Mice treated with TSA at the onset of permanent MCAo exhibited reduced infarct volume through increased Nrf2-dependent transcription of antioxidant enzymes (50). TSA also reduced transcription of inflammatory proteins MMP-1 and MMP-3, and reduced caspase-3 activation up to 24 h after the onset of ischemia in the retina (51, 52). Sodium butyrate (a potent analog of VPA) treatment before permanent MCAo evoked a 50% reduction in infarct volume through increased expression of p21, a cyclin-dependent kinase inhibitor, which prevents pro-apoptotic gene transcription (53–55). Furthermore, mice treated with 4-phenylbutyrate before or after ischemia–hypoxia exhibited reduced ER-stress-mediated apoptosis through reduction of EIF2-alpha phosphorylation (56). Mice treated with the potent hydroxamate Vorinostat (FDA approved for treatment of T-cell lymphoma) at the onset of MCAo exhibited reduced infarct size and increased transcription of Hsp70 and Bcl2 (57, 58). Furthermore, neurons exposed to oxygen–glucose deprivation *in vitro* and mice subjected to MCAo *in vivo* exhibited increased acetylation at the Bcl-xL promoter when treated with Entinostat, a class I selective HDAC inhibitor; the effect was mediated by enhanced NF-kB p50 acetylation and decreased activation of the Bim promoter (59).

While class I HDACs seem to play pathological roles in cerebral ischemia, there is evidence that class IIa HDACs are required for cell survival following neuronal stress. Genetic heterogeneity surrounding the *HDAC9* gene is associated with large vessel ischemic stroke (60). By directly inhibiting the c-jun promoter, HDAC4 (61) and HDAC7 prevented neuronal cell death induced by low potassium (62). HDAC4 is required for the normal development of retinal neurons through the stabilization of HIF-1-alpha (63). HDAC4 and HDAC5 knock-in protected neuron-like pheochromocytoma cells from apoptosis induced by OGD, which was partly dependent on HMGB1 activity (64). Conversely, nuclear export of HDAC5 was required for regeneration after acute axonal injury, a condition that promotes rapid influx of calcium (65). In fact, nuclear calcium levels regulate the association of class IIa HDACs with a MEF2-SMRT corepressor complex (66–68). Given this, it is possible that class IIa HDACs may correct calcium-induced pathological gene expression in neuronal ischemia.

## HDAC Enzymatic Crosstalk in Cerebral Ischemia

Evidence is accumulating that HDAC signal transduction pathways communicate in crosstalk with kinase signal cascades in cerebral ischemia. The ability of HDAC inhibitors to condition the neuron in the seconds to minutes following acute ischemic stress may be dependent on the concurrent activity of certain cell survival kinases. As mentioned above, TSA prevented oxidative cell death in cortical neurons through increased transcription of p21, which inactivates pro-apoptotic c-jun transcription by inhibiting the kinase ASK-1 (53–55). HDAC3 was phosphorylated by GSK3-beta and was required for cell death induced by low potassium in cultured cortical neurons; neuronal death was prevented by pharmacological inhibition of GSK3-beta, and with constitutively active Akt, a known inhibitor of GSK3-beta (69). Conversely, the class IIa HDAC4 protects neurons from cell death induced by low potassium by direct inhibition of cyclin-dependent kinase-1 activity, independent of PI3K/AKT, c-jun, or RAF/MEK/ERK signaling (61).

PI3K and AKT activities are both required for the neuronal conditioning achieved with VPA (47). Interestingly, induction of Hsp70 by VPA and other Class I HDAC inhibitors resulted in increased histone methylation in primary neurons and astrocytes (70). In particular, as confirmed by chromatin immunoprecipitation, HDAC inhibition caused increased methylation at the Hsp70 promoter, a histone landscape favoring transcriptional activation. This suggests an intricate interplay between histone acetylation and histone methylation. In fact, this phenomenon of functional and structural cooperation between HDACs and lysine-specific demethylase (LSD) enzymes is well established, as in the multifaceted corepressor CoREST/REST/HDAC/LSD complex (71). However, complex crosstalk between lysine “readers” (enzymes that recruit PTM enzymes to acetyl-lysine residues) and “writers” (enzymes that catalyze acetylation of lysine residues) results in combinations of histone modifications that form a hierarchal landscape, which dictates the transition between silencing and activation of a certain transcription domain (72). Clearly, HDAC enzymatic crosstalk with other PTM enzymes occurs on both histones and non-histone proteins.

## HDAC Inhibitors Mitigate Cardiac Infarction Following IRI

Histone deacetylase inhibitors have also shown potential in mitigating cardiac IRI (73). Importantly, HDAC activity is also upregulated in hearts after IR. Mice treated with TSA following *in vivo* IRI exhibited marked reduction of infarct area which correlated with stabilization of HIF-1a. This effect was abrogated in HDAC4 knockout cardiomycytes, in another example of the putative protective nature of a class IIa HDAC (74).

Multiple kinase pathways have been implicated in promoting myocyte survival in response to ischemic injury, including p38 MAPK (75–77), the RISK PI3K/AKT/eNOS (78–83) and RAF/MEK/ERK1/2 (84), and the survivor activating factor enhancement pathway (SAFE) (85, 86). Evidence for enzymatic crosstalk between HDACs and these pathways is growing. The cardioprotective action of HDAC inhibitors in IRI is also apparently dependent on the gp-91 subunit of NADPH oxidase (87), p38 (88), Akt1, and Mkk3 (89, 90). The transcription factor NF-kB has been suggested as a common target of multiple types of preconditioning stimuli (91). Interestingly, NF-kB is a common target of the p38 MAPK (92), Akt (93), and Erk1/2 (94) signaling pathways, and is required for p38-mediated adaptation to mechanical preconditioning in isolated hearts (92). TSA-induced pharmacological preconditioning was recently correlated with nuclear translocation, activation, and hyperacetylation of NF-kB, while specific deletion of NF-kB p50 abrogated the TSA protective effect (95). The effects of HDAC inhibition on ERK1/2 signaling have not been directly assessed in a model of IR. Intriguingly, however, HDAC inhibition prevents cell death in multiple cell lines by activating ERK1/2, while this effect is not present in several cancer cell lines (96). The absence of HDAC-mediated ERK1/2 activation in cancer cells is likely the result of suppressed HDAC1 activity (97).

Recent work in mouse tissues demonstrated that the endogenous HDAC1 inhibitor d-β-hydroxybutarate (β-OHB) caused increased acetylation at the promoter of the FOXO3a transcription factor. This led to upregulation of FOXO3a and its targets SOD2 and catalase, free radical scavengers, which further prevented paraquat-induced renal oxidative stress in mouse kidneys (98–100). Additionally, we recently reported preserved cardiac contractile function and reduced infarction following IR by pre-treating rats with Entinostat (101). This was associated with dramatic nuclear FOXO3a enrichment, along with increased transcript and protein levels of SOD2 and catalase. Nuclear enrichment of FOXO3a was likely due to decreased Akt-mediated phosphorylation at the key nuclear exclusion site S318/321 (102), suggesting that class I HDAC inhibition also influences the nuclear trafficking of transcription factors which upregulate these enzymes. HDAC4/5 are excluded from the nucleus by phosphorylation by AMPK, but localize to the nucleus in situations of low glucose, where they recruit HDAC3. Intriguingly, in this case, HDAC3 deacetylates and activates FOXO-mediated transcription of anti-apoptotic genes (103).

## Conclusion

Histone deacetylases evidently form signaling hubs for cellular communication in cerebral and cardiac IRI. Class I HDACs appear to play mainly pathologic roles in IRI, by repressing transcription of genes required for cell survival, while Class IIa HDACs appear necessary for cell survival. The roles of the class IIb HDAC6 in preconditioning are not full understood, though HDAC6 is a major regulator of autophagic flux in neurodegenerative diseases (104) and a contributor to pathological responses in the heart (105). Recent studies demonstrate that the modulation of endogenous antioxidant transcription is a significant mechanism by which the inhibition of HDACs confers preconditioning protection against IRI. Neurons and cardiomyocytes may share epigenetic signaling mechanisms for activation of endogenous protection from ischemia by HDAC inhibitors.

Lines of evidence have begun to accumulate that support a role for communication between HDAC and kinase signaling networks in ischemia of the heart–brain axis. While the mechanisms behind the protective preconditioning effect of HDAC inhibition remain to be fully elucidated, it is evident that the RISK pathway and related kinases are integral components. More research into the details of the specific interactions between HDACs and other PTMs will advance our understanding of the role of HDAC inhibition in ischemic preconditioning. Dissecting the dual roles of HDACs as transcriptional repressors and as effectors of enzymatic crosstalk is needed to dissect the chronic and acute phases of preconditioning protection. Given that several small molecule inhibitors of HDAC activity are currently used in patients or in clinical trials, HDAC inhibitors represent promising treatment modalities for patients undergoing elective brain or heart surgery, or patients at high risk of stroke or cardiac arrest.

## Conflict of Interest Statement

The authors declare that the research was conducted in the absence of any commercial or financial relationships that could be construed as a potential conflict of interest.
